# Mechanistic Insight into Diosmin-Induced Neuroprotection and Memory Improvement in Intracerebroventricular-Quinolinic Acid Rat Model: Resurrection of Mitochondrial Functions and Antioxidants

**DOI:** 10.1155/2022/8584558

**Published:** 2022-03-08

**Authors:** Mian Huang, Navpreet Singh, Ritu Kainth, Mohammad Khalid, Ajay Singh Kushwah, Manish Kumar

**Affiliations:** ^1^Department of Neurology, Wuhan Third Hospital (Tongren Hospital Affiliated to Wuhan University), No. 216, Guanshan Dadao, Wuhan, Hubei Province 430070, China; ^2^Amar Shaheed Baba Ajit Singh Jujhar Singh Memorial College of Pharmacy, Bela, Punjab 140111, India; ^3^Department of Pharmacognosy, College of Pharmacy, Prince Sattam Bin Abdulaziz University, Al-Kharj 11942, Saudi Arabia; ^4^Chitkara College of Pharmacy, Chitkara University, Rajpura, Punjab 140401, India

## Abstract

Neurodegeneration is the final event after a cascade of pathogenic mechanisms in several brain disorders that lead to cognitive and neurological loss. Quinolinic acid (QA) is an excitotoxin derived from the tryptophan metabolism pathway and is implicated in several ailments, such as Alzheimer's, Parkinson's, Huntington's, and psychosis disease. Diosmin (DSM) is a natural flavonoid possessing such properties that may halt the course of neurodegenerative progression. In past studies, free radical scavenging, along with properties, such as antihyperglycemic, anti-inflammatory, and vasoactive properties, of DSM were pragmatic. Hence, in the current experimentations, the neuroprotective activity of DSM was investigated in the QA rat prototype. QA was administered through the intracerebroventricular route (QA-ICV) in rats on day one, and DSM (50 and 100 mg/kg, intraperitoneal route) was given from day 1 to 21. Memory, gait, sensorimotor functions, and biomarkers of oxidative mutilation and mitochondrial functions were evaluated in the whole brain. Results showed significant deterioration of sensorimotor performance, gait, and working- and long-term memory in rats by QA-ICV. These behavioral anomalies were significantly attenuated by DSM (50 and 100 mg/kg) and donepezil (standard drug). QA-ICV-induced decrease in body mass (g), diet, and water ingestion were also attenuated by DSM or donepezil treatments. QA-ICV inhibited mitochondrial complex I and II activities that caused an increase in oxidative and nitrosative stress along with a reduction in endogenous antioxidants in the brain. DSM dose-dependently ameliorated mitochondrial functions and decreased oxidative stress in QA-ICV-treated rats. DSM can be a possible alternative in treating neurodegenerative disorders with underlying mitochondrial dysfunction pathology.

## 1. Introduction

Progressive neurodegeneration with concomitant cognitive and neurological deficits are the major manifestations of several brain ailments, such as Alzheimer's (AD), Parkinson's (PD), and Huntington's disease (HD). Synaptic waning and impaired long-lasting potentiation because of the decreased expression of neurotrophins (e.g., neurotrophic factors, abrineurin, and neural development factors), neurochemical aberrations (e.g., acetylcholine, glutamate, monoamines, and *γ*-aminobutyric acid), neuropeptides (e.g., oxytocin, substance P, somatostatin, and orexin), and changes in the internal milieu of the brain leads to deterioration of short term and long-term memory [1]. Excitatory pathways mediated by glutamatergic receptors are often allied with the consolidation of long-term memory in the hippocampus and cortex of the brain [2]. Receptors like *N*-methyl D-aspartate (NMDARs) are an essential component of long-lasting potentiation and depression, and calcium-influx *via* NMDARs and voltage-gated calcium (Ca^2+^) channels (VGCCs) strengthens the synapse. However, excessive excitatory drive in the brain culminates in brain atrophy *via* free radicals, proinflammatory cytokines, and activation of cell death pathways [3, 4].

Quinolinic acid (QA) is a product of the kynurenine pathway of tryptophan metabolism and is an endogenous ligand of NMDARs [5]. Although tryptophan is obligatory for serotonin and tryptamine biosynthesis, > 95% of tryptophan is metabolized through the kynurenine pathway [6]. Kynurenine pathway metabolites (e.g., kynurenic acid) are neuroactive, including QA, and they are implicated in schizophrenia, AD, and HD [7]. QA activates the immune system (microglia and astrocytes), increasing the expression of chemotactic factors (e.g., monocyte chemoattractant protein-1, RANTES) and instigating free radicals. An increase in the blood-brain barrier (BBB) penetrability prevents the shielding effect against QA, which predisposes the brain to excess QA influx. QA is a metabolic inhibitor that makes it a potent neurotoxin [8]. QA inhibits monoamine oxidase-B (MAO-B), gluconeogenesis (*via* phosphoenolpyruvate carboxykinase), creatine kinase, mitochondrial complexes, cellular respiration, and decreases ATP levels [9]. QA can augment oxidative stress and decline antioxidants in an NMDAR-dependent or -independent manner. QA-Fe^2+^ interaction instigates free radicals, leading to lipid peroxidation and DNA mutilation substantiated by an upsurge in hydroxyl radicals, poly (ADP-ribose) polymerase (PARP) activity, and lactate dehydrogenase (LDH) activity [10]. Clinical findings also revealed that QA is enhanced in the brain, blood, and cerebrospinal fluid (CSF) of AD and HD patients [5]. Findings in the past indicate that QA can induce cognitive deficits and other behavioral abnormalities in experimental animals [11]. Recent studies revealed that natural products could ameliorate the symptoms of cognitive dysfunction and improve the therapeutic outcome in neurodegenerative disorders [12, 13].

A flavonoid glycoside, diosmin (3′,5,7-trihydroxy-4′-methoxy flavone-7-rhamnoglucoside), is frequently extant in the pericarp of citrus fruits (Rutaceae) [14]. Diosmin (DSM) consists of a disaccharide group (6-*O*-(*α*-L-rhamnopyranosyl)-*β*-D-glucopyranosyl) attached with the aglycone moiety (diosmetin) through glycosidic linkage and can be biosynthesized from hesperidin. Intestinal flora transforms DSM glycoside to aglycone moiety, which is then rapidly absorbed through the gastrointestinal tract. In humans, the half-life of DSM is 26 to 43 hours when given through the oral route [15]. It is a venoactive drug that improves microcirculation, lymphatic drainage, and enhances the flexibility of veins by attenuating norepinephrine metabolism by catechol-*O*-methyl transferase. DSM abrogates microvascular permeability, leukocyte extravasation, and the appearance of adhesion molecules, such as ICAM-1 and VCAM-1 [14, 15]. Several studies indicated free radical rummaging and immune harmonizing properties of DSM in the brain [16, 17]. Clinical evidence recommends that DSM is a well-tolerable, safe, and nontoxic drug [15]. In nutraceuticals, DSM (Daflon) is often proposed to treat venous disorders, including hemorrhoids and hyperglycemic conditions. Previous findings indicated that DSM could stimulate insulin release from the *β*-cells, carbohydrate metabolism, and the expression of glucose transporters (GLUTs). Also, it decreases diabetic complications [15]. It attenuates dyslipidemia and hepatic gluconeogenesis [16]. In previous studies, DSM improved cognitive functions, attenuated the symptoms of schizophrenia, and showed neuroprotective effects in experimental animals [16–19]. Sawmiller et al. [20], in a study, noted DSM-mediated decrease in amyloid-*β* and tau hyperphosphorylation by attenuating glycogen synthase kinase 3*β* in the 3 × Tg-AD mouse model. These findings aptly signify that DSM has the potential to ameliorate brain dysfunctions against QA. In this study, QA was used to induce dementia and other neurological deficits in rats. QA can act as a potent neurotoxin that inhibits several pathways and molecular mechanisms in the brain to induce progressive neurodegeneration and brain atrophy. The contemporary investigation was designed to explore the outcomes of DSM in the QA-ICV rat prototype.

## 2. Material and Methods

### 2.1. Experimental Animals

This research was permitted by IAEC under protocol no. ASCB/IAEC/14/20/145. Albino Wistar rats (either sex, 200 g to 250 g, age 8 to 9 months old) were retained in typical size polypropylene cuboidal enclosures under artificial settings of temperature (23 ± 2°C), 12 : 12 hours dark/light sequences, and humidity (40 ± 10%) within the institutional animal house. The rodents were fed a standard nourishing foodstuff (Ashirwad Manufacturers, Punjab) and purified water at will. All animal procedures are exclusively performed as per the guidelines of CPCSEA, GOI, New Delhi. The animal custodian and handlers were blinded concerning different therapeutic regimens facilitated to animal cohorts. Investigative animal trials were executed, succeeding at least a single fortnight of familiarization duration. All investigations using animals were performed between 0900- and 1600-hours course in a day.

### 2.2. Drugs and Chemicals

Diosmin (DSM: 520-27-4), quinolinic acid (QA: 89-00-9), and standard analytes were acquired from Merck (India). Sodium dihydrogen phosphate (NaH_2_PO_4_), sodium hydroxide (NaOH), potassium phosphate dibasic (K_2_HPO_4_), nitrobluetetrazolium (NBT), phenazine methosulphate (5-methylphenazinium methyl sulphate), ethylenediaminetetraacetic acid (EDTA), bovine serum albumin (BSA), 2-[4-(2-hydroxyethyl)piperazin-1-yl]ethanesulfonic acid (HEPES), 1,2-bis[2-[bis(carboxymethyl)amino]ethoxy]ethane (EGTA), riboflavin, sodium cyanide (NaCN), natriumazid (NaN_3_), tetrasodium pyrophosphate, hydrogen peroxide (H_2_O_2_), NADH disodium (DPNH), NADPH tetrasodium (Coenzyme II reduced tetrasodium salt), phosphoric acid, Folin and Ciocalteu's phenol (FCR), and sulphosalicylic acid (5-SSA) reagent (HiMedia Laboratories, Maharashtra, India); diglycine, glacial acetic acid (CH_3_COOH), Ellman's reagent (3-Carboxy-4-nitrophenyl disulfide, DTNB), azabenzene (C_5_H_5_N), and sodium lauryl sulphate (SLS) (LobaChemie, Mumbai, India); 4,6-Dihydroxy-2-mercaptopyrimidine (2-TBA), disodium carbonate (Na_2_CO_3_), and (2-mercaptoethyl)trimethylammonium iodide acetate (TCI chemicals, India); zinc sulphate (ZnSO_4_), Rochelle salt (potassium sodium *L*(+)-tartrate), 2-(1-Naphthylamino)ethylamine dihydrochloride, nitrous acid sodium (NaNO_2_), and p-aminobenzenesulfonamide (Sisco Research Laboratories, India); butyl alcohol (Fisher Scientific, India) were used.

### 2.3. Intracerebroventricular Injection of Quinolinic Acid

Animals were subjected to anesthesia by administering intraperitoneally (*i.p.*) ketamine (90 mg/kg) and xylazine (10 mg/kg) cocktail using sterile water for injection. The body was laid in the prone position on a warm heating cushion, and in the mount of a stereotaxic surgery instrument, the head was situated. The scalp was incised at the midsagittal point, and the skull was uncovered by retracting the skin apart. Any one of the two lateral ventricles was arbitrarily chosen, and in the skull, the parietal bone was bored (stereotaxic coordinates -0.8 mm anteroposterior from bregma, ±1.5 mm mediolateral from midsagittal suture, and ±3.6 mm dorsoventral from the parietal bone surface) to make a burr hole [21]. On day one, quinolinic acid (QA) solution was freshly constituted (240 nmol) in PBS (Na^+^-K^+^ [PO_4_]^2-^ buffered saline, pH 7.4) and was gradually injected using a Hamilton microsyringe at a flow rate of 1 *µ*l/minute in the left or right cerebral ventricle of rats over 5 to 6 minutes duration with the volume of injection 5 *µ*l ICV-vehicle [22]. After the inoculation of the whole drug, the microneedle was not dislodged for 4 to 5 minutes to enable the diffusivity of the drug in the cerebrospinal fluid and thwart its regurgitation. The equivalent volume (10 *µ*l) of PBS-vehicle was administered ICV in sham rats that were identically operated, however, QA was not injected. After drug injections, the holes were restored using a luting agent (zinc phosphate, PYRAX^®^), and the stitching of the skin was accomplished. To avert contamination (bacterial growth), Neosporin^®^ was applied *pro re nata*. To evade postoperative sepsis, Orizolin (Zydus Cadila), dose 30 mg/kg (*i.p.*), was administered. Each rat was provided a warm environment (37 ± 0.5°C) to avert postsurgical hypothermia. Each rat was allowed semisolid food (inside the cage) and water *gratis* after surgery for seven days and housed discretely in a distinct cage (30 × 23 × 14 cm^3^).

### 2.4. Experimental Protocol

**Figure 1:**
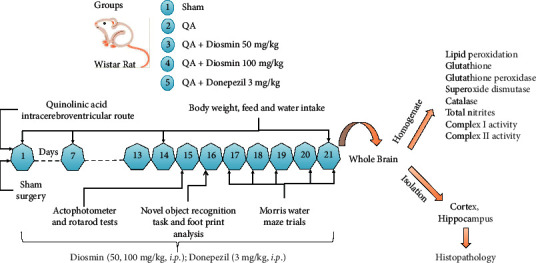
Diosmin (DSM) was administered (dose 50 and 100 mg/kg *b.w.*) for 21 days after intracerebroventricular (ICV) quinolinic acid (QA; 240 nmol) treatment on day 1. Bodyweight, food, and water intake were observed daily and analyzed weekly. The locomotor function and sensorimotor performance of animals were assessed on day 15 using an actophotometer and rotarod apparatus. The working type discriminative memory was gauged in rats on day 16 using a novel object recognition test (NORT), and subsequently, all animal clusters were subjected to footprint analysis. Training trials in the Morris water maze (MWM) test were given from day 17 to 20, and retention trials were conducted on day 21. Afterward, whole brains were secluded to evaluate the biochemical parameters of mitochondrial functions and oxidative stress.

DSM was injected at doses 50 and 100 mg/kg per body weight (*b.w.*) in rats through the intraperitoneal (*i.p.*) route using 0.5% dimethylsulfoxide vehicle in normal saline (dose-volume 5 ml/kg) [17]. Animals were randomly allocated in 5 clusters in a single-blind mode (*n* = 5): (i) sham (S), (ii) QA, (iii) QA + DSM50, (iv) QA + DSM100, and (v) QA + DNP. Rats were subjected to the intracerebroventricular administration of QA (QA-ICV) or sham surgery on the 1^st^ day. DSM was administered for 21 consecutive days daily 120 minutes after QA-ICV from day one onwards. Donepezil (DNP) was employed as a standard drug in this study and injected (dose 3 mg/kg, *i.p.*) in QA-ICV-injected rats for 21 successive days. Animals in the sham and QA control groups were administered vehicle (sterile 0.5% dimethylsulfoxide in normal saline in dose-volume 5 ml/kg) from day 1 to 21. The whole study was performed according to the scheme depicted in Figure 1.

### 2.5. Locomotor Activity

In all rat clusters, the mean locomotor activity was documented by means of an actophotometer device for 5 minutes. A separate animal was positioned in the actophotometer for 3 minutes of acclimatization. The rats were then given 5 minutes, and the results were stated as counts per 5 minutes [11].

### 2.6. Rotarod Test

In rodents, the rotarod test typically evaluates the equilibrium and muscle synchronization facets of sensorimotor functions. The rats were presented to acquisition trials until their ability to run reached >60 seconds on the rod revolving at nine rotations per minute (rpm). After the acquisition trials, a separate rat was positioned on the cylindrical shaft, and the revolution velocity was boosted at a constant intermission of 10 seconds from 6 rpm (preliminary speed) to 30 rpm (concluding speed), spanning over 50 seconds. The mean fall-off latency (in seconds) from the revolving cylindrical shaft was stated in the results.

### 2.7. Footprint Analysis

The principle behind performing footprint analysis in rats is assessing the gait abnormalities. For footprints, rat feet were immersed in four diverse colored nontoxic food dyes and were permitted to run on an inclined walkway (70 cm × 10 cm × 8 cm). The runway base was enclosed with a cellulose sheet of white color. The rats were motivated to a dim uphill section at the end of the runway to obtain clear footprints. The dye was gently removed from each animal using lukewarm water after the trials. The footprints were scanned, and the “stride length” was measured by means of a standard ruler. Stride length was quantified by calculating the distance between the sequential placements of the identical rat's paw [11].

### 2.8. Novel Object Recognition Task (NORT)

Standard protocol was followed as given by Kumar and Bansal [23]. NORT is an unprofitable and nonhostile exteroceptive archetype employed to assess the working type recollective memory exploited through the impulsive probing conduct of rodents. The investigation is performed in a rooftop-open plywood cuboidal vessel (80 cm × 42 cm × 62 cm), positioned in a quiet area, illuminated by a 60W LED to manage consistent brightness in the vessel. Cylinder (white)-, pyramid (red)-, and cube (black)-shaped (12 cm tall) wooden items (in identical triplets) were solid and of enough weight to render them immobile by rodents. NORT was performed on the 16^th^ day in 3 stages (S): acclimatization (S1), acquisition (S2), and novel object recognition examination (retention) stage (S3). During S1, three successive days before the trials were issued to discover the vacant floor base of the vessel (5 minutes) by the rats. Upon the completion of S1, the individual animal was habituated to any one set of solid items in the learning stage (S2). Twin alike things were positioned in 2 arbitrarily selected contrary angles of the vessel (9 cm to 11 cm gap from the side ramparts). Separately, a rodent was positioned at the center of the vessel facing opposite to the two solid items and was permitted to discover the two similar items for 5 minutes. Guiding the snout near the object at ≤2–3 cm distance or physical contact with the item with the muzzle was supposed as investigative conduct. After S2, the rodent was housed in a home cage trailed by an intertrial recess (ITR) of 60 minutes. Any single solid item offered in S2 was swapped by a different solid item, and the rodents were presented again to the twin items, i.e., a replica of the acquainted item and the different item. The whole of the amalgamations and positions of the items were offset to abate likely prejudice instigated by a penchant for certain settings or items. The vessel and solid items were meticulously wiped (ethyl alcohol 15% and dry cloth) after every investigation to curb the odorous signs. The period expended discovering each item in S2 and S3 was documented using a stopwatch. The duration expended investigating the two matching items in S2 (*I*1 = *I*i1 + Ii2) and the duration expended investigating the two dissimilar items, i.e., acquainted and different, in S3 (*I*2 = *Ii*3 + *Ib*) was recorded. The variance in duration expended investigating the different item and the duration of investigating the acquainted item (*Ib*–*Ii*3 = DI) discloses the retention of recollective memory. DI (discrimination index)/S3 duration (*s*) of investigating both the acquainted and new item (amended DI) improves the partialities by variances in the complete investigation and denotes the penchant for different items in contrast to acquainted ones {DI = (*Ib*–*I*i3)/(*Ii*3 + *Ib*)}. Recollective memory was appraised by quantifying the skill of rodents to single out the familiar/novel items in S3 and was stated as DI (amended for the overall investigation period in S3) [24].

### 2.9. Morris Water Maze (MWM)

The standard protocol was followed, as given by Kumar and Bansal [25]. MWM judges the spatial memory by swimming trials, in which the rodent finds an escape route to a concealed podium. A black colored circular tank (2 m diameter, 0.6 m height) had water (25 ± 1°C) filled to a depth of 0.3 m. This aquatic reservoir was separated clockwise into 4 similar regions (R1, R2, R3, and R4) using two nylon fibers, secured perpendicularly on the upper perimeter of the tank. A dais (10.5 cm × 10.5 cm) was positioned underwater (1 cm underneath water) in the reservoir region R4. The spot of the dais persisted intact all over the acquisition period. Every single rat was presented with four serial acquisition rounds (5 minutes ITR) every day. The rodent was gently released into the aquatic reservoir facing the tank wall, with the site varying with every single trial from R1-R4, R2-R1, R3-R2, and R4-R3 on days 1 to 4, respectively, and it was permitted 120 seconds to detect the underwater podium. The rodents continued to rest on the podium for 20 seconds. A failure to detect the platform within 120 seconds indicated the manual placement of rats on the platform, and then they were permitted 20 seconds on the platform. Escape latency time (ELT) is the duration (*s*) of discovering the concealed dais in the aquatic reservoir. Spatial learning was marked by day one *vs.* day four ELT. In the probe trial (5^th^ day), the rodents investigated the reservoir for 120 seconds but were deprived of the podium. The mean duration expended in the entire reservoir (4 regions) was recorded. The mean duration expended in R4 (TSTQ: time spent in target quadrant) probing for the concealed podium was deemed as an index of reference memory. The comparative setting of the tank relative to the items in the laboratory that act as visual signs and the investigator's position remained undisturbed [26].

### 2.10. Estimation of Biochemical Parameters

After completing the behavioral examinations, the complete brain of the rats was garnered and positioned on pulverized ice cubes, followed by bathing with freezing sterilized saline (isotonic 308 mOsmol/l NaCl) to remove the remains and blood. Homogenization of the entire brain was instantly accomplished in a freezing separation buffer (pH 7.4) with the composition 215 mM D-mannitol, 20 mM 2-[4-(2-hydroxyethyl)piperazin-1-yl]ethanesulfonic acid, 1 mM 1,2-bis[2-[bis(carboxymethyl)amino]ethoxy]ethane, 75 mM saccharose, and 0.1% BSA. The homogenate was centrifugated at 4°C using 13000 × g force for 5 minutes. The pellet was rejected, and the supernatant was separated into two portions and was recentrifuged (4°C) at 13000 × g force for 5 minutes. The crude mitochondrial pellet was separated and again centrifuged in a separation buffer with 1,2-bis[2-[bis(carboxymethyl)amino]ethoxy]ethane at 12,500 × g for 11 minutes (4°C). The semisolid deposit so obtained comprising uncontaminated mitochondria was resuspended in a separation buffer (pH 7.4) containing 75 mM saccharose, 20 mM 2-[4-(2-hydroxyethyl)piperazin-1-yl]ethanesulfonic acid, and 215 mM D-mannitol [27]. Later, the mitochondrial fraction of the whole-brain homogenate was used to determine the biochemical markers using standard methods.

### 2.11. Estimation of Mitochondrial Complex

#### 2.11.1. NADH: Ubiquinone Oxidoreductase Activity

The rate of complex I (NADH dehydrogenase) activity was quantified (nmol NADH oxidized/minute/mg protein) by following the technique of King and Howard [28]. The oxidative generation of NAD^+^ from NADH is accompanied by cytochrome *c* reduction. The assay blend consisted of cytochrome *c* (10.5 mM), 6 mM *β*-nicotinamide adenine dinucleotide (DPNH) dissolved using 2 mM diglycine buffer, and diglycine buffer (0.2 M, pH 8.5). A dissolvable mitochondrial fraction was incorporated in the assay concoction to trigger the reaction. The variation in optical density (O.D.) at *λ*_max_ = 550 nm was followed for 120 seconds.

#### 2.11.2. Succinate: Ubiquinone Oxidoreductase Activity

The rate of succinate dehydrogenase (complex II) activity was quantified (nmol succinate oxidized/minute/mg protein) by following the technique of King [29]. Succinic acid oxidation is triggered by a mock electron receiver, potassium cyanoferrate (K_3_Fe(CN)_6_). The assay blend comprised of succinic acid (0.63 M), 1% BSA, K_3_Fe(CN)_6_ (0.036 M), and Na^+^-K^+^ [PO_4_]^2-^ buffer (0.23 M, pH 7.6). A dissolvable mitochondrial fraction was incorporated in the assay concoction to trigger the reaction. The variation in O.D. at *λ*_max_ = 420 nm was followed for 120 seconds.

### 2.12. Determination of Oxidative Stress Biomarkers

#### 2.12.1. Thiobarbituric Acid Reactive Substances (TBARS)

To evaluate TBARS (nmol per mg protein) [30], the analyze combination (concluding quantity ∼4 ml) comprising 0.10 ml homogenized brain, 1.51 ml 4,6-dihydroxy-2-mercaptopyrimidine (0.8%), 200 *µ*l SLS (8.18%), 1.49 ml glacial acetic acid (21%, pH 3.51), and 0.71 ml deionized water was subjected to water-bath heating at 96°C for 60 minutes. A 15 : 1 ratio butyl alcohol/azabenzene (5.1 ml) was supplemented in analyze concoction that was centrifugated at 4,000 × g power (10 minutes), and the supernatant was secluded. With a twin-beam UV1700 spectrophotometer (Shimadzu, Japan), chromophore malondialdehyde-4,6-dihydroxy-2-mercaptopyrimidine O.D. was appraised at a wavelength (*λ*_max_ = 532 nm), and *ε* = 1.56 × 10^5^/M/cm (molar extinction coefficient) was applied to compute 4,6-dihydroxy-2-mercaptopyrimidine adducts.

#### 2.12.2. Reduced Glutathione (L-*γ*-Glutamyl-L-Cysteinyl-glycine) Levels

Ellman's [31] procedure was implemented to appraise L-glutathione (GSH) content. The test concoction encompassing homogenate (1.1 ml) and 1 ml of 4% 2-hydroxy-5-sulfobenzoic acid (5-SSA) was centrifugated (4°C) for 11 minutes at 2,500 × g power. Later, 2.8 ml Na^+^-K^+^ [PO_4_]^2-^ buffer (51.2 mM, pH 7.77) and 0.21 ml 3-carboxy-4-nitrophenyl disulfide (0.12 mM, pH 7.89) was blended with the above-separated supernatant (0.12 ml). Tripeptide (*µ*mol GSH per mg protein) was quantified with the twin-beam UV1700 spectrophotometer (*λ*_max_ = 412 nm). applying *ε* = 1.36 × 10^4^/M/cm.

#### 2.12.3. Glutathione Peroxidase Activity

The activity of glutathione peroxidase (GPx) (EC 1.11.1.9) was appraised by implementing the technique of Mohandas et al. [32]. The analyze blend comprised of 100 *µ*l of 10% homogenate, 100 *µ*l sodium azide (1.11 mM), 100 *µ*l EDTA (1.13 mM), 40 *µ*l glutathione-disulfide reductase (GSR, 1 IU/ml) (EC 1.8.1.7), 10 *µ*l H_2_O_2_ (0.28 mM), 40 *µ*l L-*γ*-glutamyl-L-cysteinyl-glycine (1.2 mM), 100 *µ*l coenzyme II reduced tetrasodium salt (0.22 mM), and 0.12 M 1.49 ml Na^+^-K^+^ [PO_4_]^2-^ buffer (pH 7.4) in an entire quantity of 2000 *µ*l. The loss of coenzyme II-reduced tetrasodium at *λ*_max_ = 340 nm was documented at a temperature of 25°C. GPx rate was computed as nmol NADPH oxidized/minute/mg protein by means of *ε* = of 6.22 × 10^3^/M/cm.

#### 2.12.4. Superoxide Dismutase Activity

The rate of SOD (EC 1.15.1.1) action (units per mg protein) was considered by the procedure of Kakkar et al. [33]. The reaction concoction involved 0.3 ml homogenate, 100 *µ*l 5-methylphenazinium methyl sulphate (197 *μ*M), and 1.3 ml sodium diphosphate tetrabasic (0.066 mM, pH 7.2). The reaction was commenced using 200 *µ*l of *β*-nicotinamide adenine dinucleotide (DPNH) (780 *μ*M) and halted 60 seconds later using 1 ml glacial CH_3_COOH in this blend. Chromogen quantity generated was computed by noting the color strength at *λ*_max_ = 560 nm.

#### 2.12.5. Catalase Activity

To assess the rate of catalase (EC 1.11.1.6) action, the O.D. discrepancy (*λ*_max_ = 240 nm) of the analyze concoction (3.0 ml) comprising 50 *µ*l investigating sample, 1.22 ml H_2_O_2_ (0.03 M) in Na^+^-K^+^ [PO_4_]^2-^ buffer (pH 7.91, 0.06 M), and 1.63 ml of 0.06 M Na^+^-K^+^ [PO_4_]^2-^ buffer (pH 7.1) was recorded. Catalase activity (*µ*mol H_2_O_2_ decayed per minute per mg protein of brain) was computed by applying *ε* = 43.6/M/cm [34].

#### 2.12.6. Whole Nitrites Level

The technique of Sastry et al. [35] was implemented to evaluate entire brain nitrites (*μ*mol per mg of brain protein). In test tubes encompassing 100 *µ*l investigative sample, 145 mg amalgam of copper–cadmium, 500 *µ*l H_2_CO_3_ buffer (pH 8.89), 0.44 M 100 *µ*l NaOH, and 119.8 mM 400 *µ*l ZnSO_4_ were centrifugated at 4,500 × g power for 10 minutes, and the superfluous upper liquid (supernatant) was secluded. Griess chemical (50 *µ*l) was included in 100 *µ*l of *s* superfluous liquid. After 60 minutes of incubation, O.D. (*λ*_max_ = 548 nm wavelength) was noted employing a twin-beam UV1700 spectrophotometer (Shimadzu). A typical curve of nitrous acid sodium (0.02–0.2 mM) was designed, and the entire nitrite was equated.

#### 2.12.7. Determination of Total Proteins

The overall protein level (mg/ml of homogenate) was computed by means of a typical curvature graph of bovine serum albumin with the solution strength ranging from 0.3 to 3.8 mg/ml. The examination combination was organized with 250 *µ*l homogenate, 5.1 ml Lowry's reagent, Na^+^-K^+^ [PO_4_]^2-^ buffer (900 *µ*l), and 1.1 N 500 *µ*l FCR. The discrepancy of O.D. was observed at *λ*_max_ = 650 nm [36].

### 2.13. Brain Sections Histopathology

By means of a gravity-fed diffusion setup, rats were intracardially (*via* left ventricle) diffused with 10% neutral buffered formaldehyde (10% NBF) solution and acutely anesthetized. *Hippocampus* and cortical sectors are immersed in a fixative (10 : 1 fixative: tissue proportion), namely 10% NBF for one week (4°C), accompanied by 0.04% natriumazid (pH 7.4). Ethyl alcohol (70%) was employed as a packing solution for fixed tissue portions kept at 4°C. A microtome cutter (rotation type) was employed to acquire thin portions (8.0 *μ*m), which were then tinted with colorant hematoxylin and eosin (H&E). The slides were made permanent by means of DPX-resin, which were later cover-slipped and inspected through an optical microscope (binocular) at ×40 magnifications.

### 2.14. Statistical Analysis

A skilled experimenter blinded to miscellaneous drug regimens given to animal cohorts scrutinized and evaluated the data. Outliers were not pragmatic (Grubb's test) in the data, and the Kolmogorov–Smirnov test and Levene's test confirmed the normal distribution of variables and homogeneity of variance (HOV *p* > 0.05, Levene's test), respectively. Otherwise, in case of unequal variance (HOV *p* < 0.05, Levene's test), Welch's ANOVA (*p* < 0.05, F′-statistic) and Games–Howell post hoc tests can be applied. The means of normally distributed variables were scrutinized and related by one-way or repeated measures of two-way analysis of variance (ANOVA). In the case of ANOVA, outcomes are significant (*p* < 0.05) in F-statistics, multiple comparison tests, namely Tukey's HSD (honest significant difference) or Bonferroni, were applied. Statistical significance was deemed at *p* < 0.05, and the results were stated as mean ± Standard Error of Mean (SEM).

## 3. Results

### 3.1. Outcomes of DSM on Body Mass (g), Diet, And Water Ingestion of Rats Administered QA-ICV

**Figure 2:**
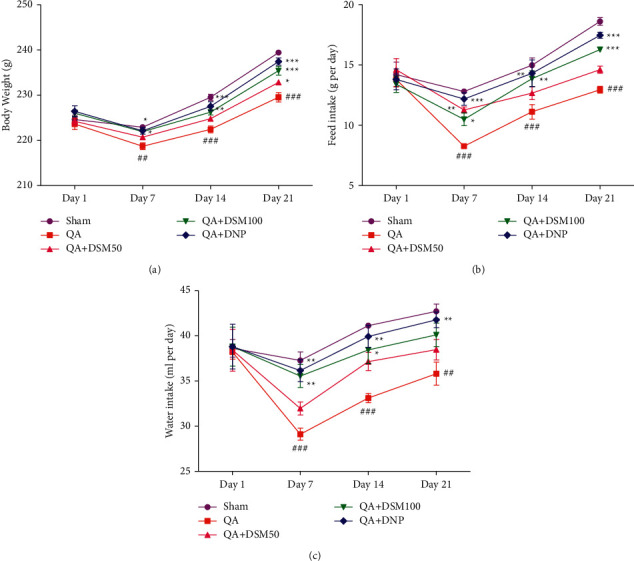
Outcomes of diosmin (DSM) treatment (doses 50 and 100 mg/kg) for 21 repeated days on the body mass (g), diet, and water consumption of rats exposed to QA-ICV on 1^st^ day. Statistical scrutiny of (a) body weight (g), (b) diet ingestion (g), and (c) water consumption (ml) was done each week using repeated measures of two-way ANOVA and Bonferroni post hoc test. Data are presented as mean ± SEM (*n* = 5). ^###^*p* < 0.001 *vs.* Sham; ^*∗*^*p* < 0.05, ^*∗∗*^*p* < 0.01, ^*∗∗∗*^*p* < 0.001 *vs.* QA group. Body weight: [*F*_(12,80)_ = 3.54, *p* < 0.001], feed intake: [*F*_(12,80)_ = 3.04, *p* < 0.001], water intake: [*F*_(12,80)_ = 1.30, *p* < 0.001].

Body weights, feed, and water ingestion were analyzed weekly, starting from day 1. A significant reduction (*p* < 0.001) in body mass (g), feed, and water intake on day 7, 14, and 21 was pragmatic in rats subjected to QA-ICV injection on day 1 when compared to sham counterparts (Figure 2). DSM (100 mg/kg) dosing caused a significant increase in the body weight (day 7 *p* < 0.05, day 14 *p* < 0.01, day 21 *p* < 0.001), feed (day 7 *p* < 0.05, day 14 *p* < 0.01, day 21 *p* < 0.001), and water intake (day 7 *p* < 0.01, day 14 *p* < 0.05) in rats against QA-ICV. DSM (50 mg/kg) also significantly attenuated QA-ICV-triggered decrease in the body weight (day 21 *p* < 0.05) and feed intake (day 7 *p* < 0.01) of rats relative to rats that have lone QA-ICV injections. QA-ICV- and DNP-treated rats disclosed a significant escalation in body mass (g) (day 7 *p* < 0.05, day 14 *p* < 0.001, day 21 *p* < 0.001), feed (day 7 *p* < 0.001, day 14 *p* < 0.01, day 21 *p* < 0.001), and water intake (day 7, 14, 21 *p* < 0.01) relative to rats that remained exposed to lone QA-ICV.

### 3.2. Effect of DSM on Locomotion, Motor Coordination, and Gait of Rats against QA-ICV

**Figure 3:**
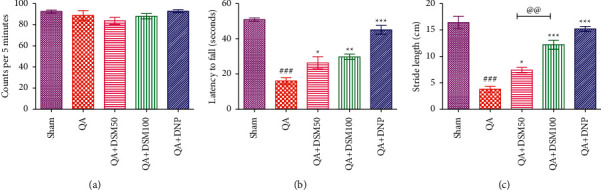
Effect of diosmin (DSM) treatment (doses 50 and 100 mg/kg) on locomotor activity, motor coordination (sensorimotor performance), and the gait of rats exposed to QA-ICV on the first day. Statistical analysis of (a) locomotor activity using actophotometer on day 15, (b) sensorimotor performance using rotarod apparatus on day 15, and (c) gait using foot print analysis on day 16 was done employing one-way ANOVA and Tukey's HSD post hoc test. Data are presented as mean ± SEM ((n) = 5). ^###^*p* < 0.001 *vs.* Sham group; ^*∗*^*p* < 0.05, ^*∗∗*^*p* < 0.01, ^*∗∗∗*^*p* < 0.001 *vs.* QA group; ^@@^*p* < 0.01 QA + DSM100 *vs.* QA + DSM50. Locomotor activity: [*F*_(4,24)_ = 1.62, (p) > 0.05], motor coordination: [*F*_(4,24)_ = 41.03, *p* < 0.001], stride length: [*F*_(4,24)_ = 46.48, *p* < 0.001].

In this study, the locomotor activities of animals were not affected by QA-ICV or drug treatments. QA group depicted no significant change in the mean counts per 5 minutes in the actophometer apparatus in comparison to the sham group (Figure 3(a)). Rotarod and footprint analysis were used to evaluate the sensorimotor performance and gait of rats. Results showed that QA-ICV significantly hampered (*p* < 0.001) motor coordination (Figure 3(b)) and gait (Figure 3(c)) of rats, reflected by a decrease in latency to fall from the revolving shaft and the stride length of rats in footprint analysis in comparison to sham counterparts. QA + DSM50 and QA + DSM100 groups portrayed a noteworthy increase in the falling latency (*p* < 0.05, *p* < 0.01) and stride length (*p* < 0.05, *p* < 0.001) relative to QA group. DNP treatment significantly attenuated QA-ICV triggered decrease in latency to fall (*p* < 0.001) and stride length (*p* < 0.001) when compared to rats that were given QA-ICV alone. Furthermore, DSM (100 mg/kg) treatment displayed a significant improvement (*p* < 0.01) in gait relative to DSM (50 mg/kg) in rats subjected to QA-ICV.

### 3.3. Effect of DSM on Working Memory and Spatial Long-Term Memory of Rats against QA-ICV

**Figure 4:**
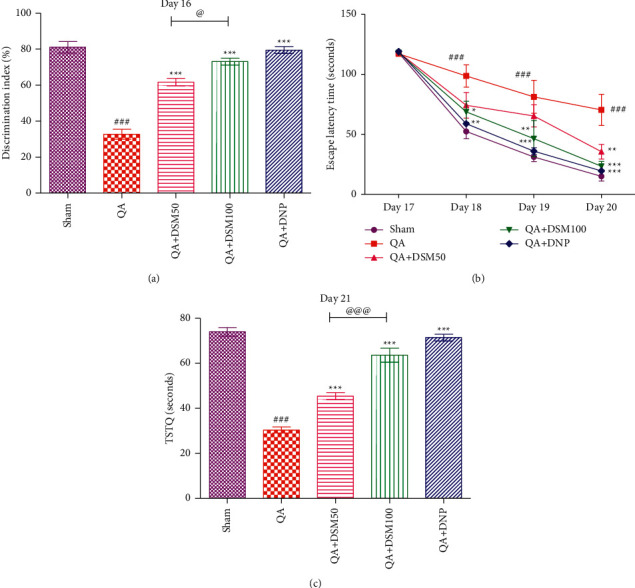
Effect of diosmin (DSM) treatment (doses 50 and 100 mg/kg) on working memory and spatial long-term memory of rats against QA-ICV was evaluated using novel object recognition task (NORT) and Morris water maze (MWM), respectively. Statistical analysis of (a) discrimination index (%) in NORT, (b) escape latency time (ELT) in training trials using MWM, and (c) time spent in target quadrant (TSTQ) in retention trials using MWM. One-way ANOVA and Tukey's HSD post hoc test was used for statistical analysis. For time course data (ELT) repeated measures of two-way ANOVA and Bonferroni *post-hoc* test were used. Data are presented as mean ± SEM ((n) = 5). ^###^*p* < 0.001 *vs.* Sham group; ^*∗*^*p* < 0.05, ^*∗∗*^*p* < 0.01, ^*∗∗∗*^*p* < 0.001 *vs.* QA group; ^@^*p* < 0.05, ^@@@^*p* < 0.001 QA + DSM100 *vs.* QA + DSM50. DI(%): [*F*_(4,24)_ = 68.15, *p* < 0.001], ELT: [*F*_(12,80)_ = 2.21, *p* < 0.001], TSTQ: [*F*_(4,24)_ = 86.2, *p* < 0.001].

A decrease in discrimination index (%) in NORT corroborated a decrease in the working type memory. A gradual increase in ELT over 4 days of training trials and a decrease in TSTQ in the retrieval trials (conducted 24 hours after last training trial) in MWM denoted long-term memory loss in rats. In this study, rats that were exposed to QA-ICV treatment alone indicated a noteworthy decline (*p* < 0.001) in the discrimination index (%) relative to sham (Figure 4(a)). In the MWM test, day 17 training trials revealed no significant alteration in ELT among different groups, however, on day 18, marked change in ELT was noted. QA cohort displayed a substantial (*p* < 0.001) increase in ELT (day 18–20) (figure 4(b)) and decrease in TSTQ (day 21) (Figure 4(c)) relative to sham counterparts. Treatment with DSM (100 mg/kg)-attenuated QA-ICV prompted diminution in discrimination index (%) (*p* < 0.001), increase in ELT (day 18 *p* < 0.05, day 19 *p* < 0.01, day 20 *p* < 0.001), and decrease in TSTQ (*p* < 0.001) when compared with the rats that have undergone QA-ICV injection alone. QA + DSM50 cohort exhibited a substantial upsurge in discrimination index (%) (*p* < 0.001), decrease in day 20 ELT (*p* < 0.01), and increase in TSTQ (*p* < 0.001) relative to the QA group. DNP treatment enhanced the discrimination index (%) (*p* < 0.001), decreased ELT (day 18 *p* < 0.01, day 19 *p* < 0.001, day 20 *p* < 0.001), and increased TSTQ (*p* < 0.001) in rats that were administered QA-ICV in comparison to vehicle-treated QA-ICV rats. Furthermore, DSM (100 mg/kg)-repeated injections disclosed a substantial improvement in memory functions relative to DSM (50 mg/kg) in rats subjected to QA-ICV.

### 3.4. Outcomes of DSM on Brain Mitochondrial Complex in QA-ICV Injected Rats

**Figure 5:**
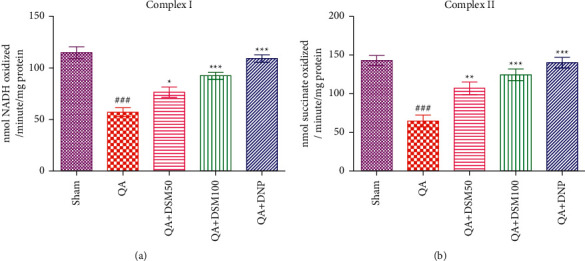
Effect of diosmin (DSM) treatment (doses 50 and 100 mg/kg) on mitochondrial complex of the brain of rats against QA-ICV. Statistical analysis of (a) complex I (NADH dehydrogenase), (b) complex II (succinate dehydrogenase) using one-way ANOVA, and Tukey's HSD post hoc test. Data are presented as mean ± SEM (*n* = 5). ^###^*p* < 0.001 *vs.* Sham, ^*∗*^*p* < 0.05, ^*∗∗*^*p* < 0.01, ^*∗∗∗*^*p* < 0.001 *vs.* QA group. Complex (I) [*F*_(4,24)_ = 27.45, *p* < 0.001], Complex II: [*F*_(4,24)_ = 19.17, *p* < 0.001].

Mitochondrial activity in the whole-brain homogenate was evaluated after behavioral trials. Results showed a significant decline (*p* < 0.001) in the complex I/II rates in the mitochondrial fraction of the brain homogenate by QA-ICV relative to sham (Figure 5). This decrease in complex I/II activity by QA-ICV treatment was attenuated (complex I *p* < 0.05, *p* < 0.001; complex II *p* < 0.01, *p* < 0.001) by DSM (50 and 100 mg/kg) given for 21 successive days in comparison to QA-ICV-administered rats that were given drug vehicle treatment alone. DNP significantly enhanced (*p* < 0.001) the activity of complex I/II in contrast to the vehicle in QA-ICV injected rats.

### 3.5. Effect of DSM on Brain Mitochondrial Oxidative Stress in QA-ICV Injected Rats

**Figure 6:**
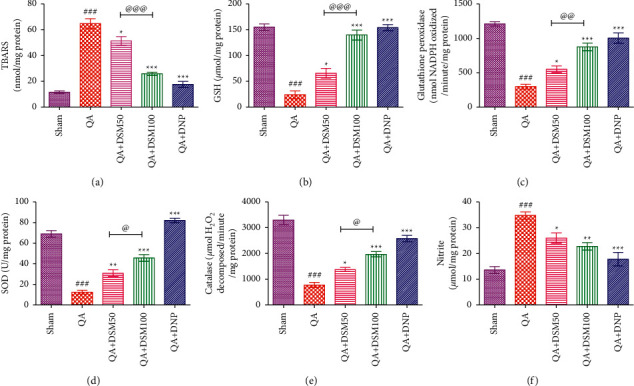
Effect of diosmin (DSM) treatment (doses 50 and 100 mg/kg) on the brain mitochondrial oxidative and nitrosative stress in rats against QA-ICV. Statistical analysis of (a) lipid peroxidation (TBARS), (b) glutathione (GSH), (c) glutathione peroxidase (GPx) activity, (d) superoxide dismutase (SOD) activity, (e) catalase activity, and (f) total nitrites using one-way ANOVA and Tukey's HSD post hoc test. Data are presented as mean ± SEM (*n* = 5). ^###^*p* < 0.001 *vs.* Sham group; ^*∗*^*p* < 0.05, ^∗∗^*p* < 0.01, ^*∗∗∗*^*p* < 0.001 *vs.* QA group; ^@^*p* < 0.05, ^@@^*p* < 0.01, ^@@@^*p* < 0.001 QA + DSM100 *vs.* QA + DSM50. TBARS: [*F*_(4,24)_ = 75.99, *p* < 0.001], GSH: [*F*_(4,24)_ = 56.57, *p* < 0.001], GPx: [*F*_(4,24)_ = 49.46, *p* < 0.001], SOD: [*F*_(4,24)_ = 98.94, *p* < 0.001], catalase: [*F*_(4,24)_ = 63.83, *p* < 0.001], nitrites: [*F*_(4,24)_ = 19.76, *p* < 0.001].

Results exhibited a noteworthy increase (*p* < 0.001) in the TBARS and total nitrites and a decline in GSH, GPx, SOD, and catalase activities in the mitochondrial fraction of the brain homogenate by QA-ICV relative to sham (Figure 6). This augmentation in the brain TBARS (*p* < 0.05, *p* < 0.001) and total nitrites (*p* < 0.05, *p* < 0.01) and decline in GSH (*p* < 0.05, *p* < 0.001), GPx (*p* < 0.05, *p* < 0.001), SOD (*p* < 0.01, *p* < 0.001), and catalase (*p* < 0.05, *p* < 0.001) activities by QA-ICV treatment was attenuated by DSM (50 and 100 mg/kg) given for 21 uninterrupted days in comparison to QA-ICV-administered rats that were given drug vehicle treatment only. DNP significantly depriciated (*p* < 0.001) brain TBARS and total nitrites accumulation and enhanced (*p* < 0.001) the activity of GSH, GPx, SOD, and catalase in comparison to the vehicle in QA-ICV-treated rats. Furthermore, DSM (100 mg/kg) treatment displayed a noteworthy depreciation in lipid peroxidation (*p* < 0.001) and intensification in endogenous antioxidants, such as GSH (*p* < 0.001), GPx (*p* < 0.01), SOD (*p* < 0.05), and catalase (*p* < 0.05), relative to DSM (50 mg/kg) in rats subjected to QA-ICV.

### 3.6. Effect of DSM on Brain Histopathology in Rats against QA-ICV

**Figure 7:**
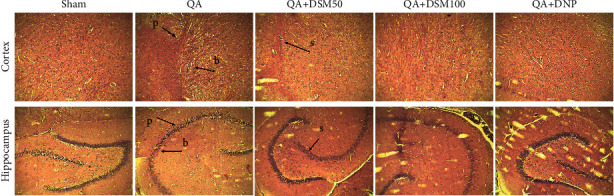
Effect of diosmin (DSM) treatment (doses 50 and 100 mg/kg) on QA-ICV prompted neurodegenerative deviations in the cortical and hippocampus sections (*n* = 5) (H&E stain, × 40, scale 10 *µ*m). Pyknosis (p), bulging of the plasma membrane (b), and swelling (s) were observed.

In histopathology analysis, major changes in the cellular architecture were observed in the QA group. Sham animals showed no signs of neurodegeneration. QA-ICV treatment caused marked changes highlighted by pyknosis and the blebbing of the plasma membrane in the cortical and the hippocampus (CA 1 and (2) neurons. Treatment of QA-ICV rats with DSM or DNP attenuated the pathological signs of neurodegeneration (Figure 7).

## 4. Discussion

QA (2,3-pyridine dicarboxylic acid) is an excitotoxin similar to glutamate and is capable of evoking neurodegeneration as its concentration amplifies with age [5]. The antagonists of NMDARs and amino phosphonates can prohibit the neurodegenerative excitotoxicity of QA, which suggests that QA acts through NMDARs in the brain [9]. BBB acts as a protective barrier and limits the neurotoxicity of QA. However, the pieces of evidence indicate enhanced pathological accumulation of QA in diverse neurodegenerative disorders. In the brain, quinolinate phosphoribosyltransferase (QPRT) catabolizes QA to NAD^+^ and carbon dioxide. The activity of QPRT is maximum in the olfactory bulb and lowermost in the cortex, hippocampus, and striatum, where QA may exert neurotoxic action to a great extent in these brain regions. These brain areas are adversely affected in numerous neurodegenerative disorders, such as AD, PD, HD, and schizophrenia that lead to severe cognitive decline [9, 10]. In this study, QA was administered directly through the ICV route to overcome the BBB constraint in the adult rats. DSM is a bioactive natural flavonoid that has shown therapeutic effects against traumatic brain injury [16], scopolamine-induced amnesia [17], apomorphine- and ketamine-induced psychosis [18], and chronic unpredictable mild stress [19]. DSM is capable of enhancing glucose metabolism, insulin signaling, and diabetic complications, and it may prevent energy depletion and the ensuing adverse consequences implicated in neurodegenerative disorders [14, 15]. QA prohibits mitochondrial function and energy-producing aerobic respiratory pathways in the brain regions adversely affected in neurodegenerative disorders [5]. Hence, in the present study, rats administered with QA-ICV on day one were exposed to DSM treatment for 21 consecutive days, and biochemical parameters and behavioral functions were assessed.

The findings of this study indicated an increase in oxidonitrosative stress and depreciation of endogenous antioxidant levels by QA-ICV in the mitochondrial fraction of the whole-brain homogenate. Previous studies also indicate NMDAR-dependent and NMDAR-independent increase in free radicals and inflammatory deterioration by QA in experimental animals [5, 9, 10]. In current experiments, QA enhanced lipid peroxidation and total nitrites in the brain. Free radicals and the ensuing modifications in cellular biomolecules, such as lipids, proteins, and DNA, underlie major pathogenic changes in neurodegenerative disorders. Lipid peroxides, such as malondialdehyde (MDA), 4-hydroxy 2-nonenal (4-HNE), isoprostanes, and acrolein are highly toxic aldehydes that readily accumulate in the form of bio-adducts and are resistant to autophagic and other mechanisms of removal [37, 38]. A pathogenic rise in these insoluble adducts breaches the integrity of the cell, which leads to a loss of internal homeostasis, leakage of internal components, and cell death [38]. Furthermore, an increase in nitrates directly correlates with the level of nitric oxide release in the brain of rats. The administration of QA-ICV on the first day instigated a noteworthy escalation in nitrates in the brain of rats. Nitric oxide is a gaseous neurotransmitter that participates in synaptic modulation and long-term potentiation by acting in a rearward manner through NMDARs [39, 40]. Nitric oxide is biosynthesized by nitric oxide synthase (NOS- neuronal) in response to the activation of postsynaptic NMDARs. An influx of calcium ions through postsynaptic NMDARs activates neuronal NOS, leading to the generation of nitric oxide that stimulates presynaptic glutamate release in the synapse. However, excessive NMDAR activation and the resulting overt intracellular influx of calcium ions and nitric oxide biosynthesis leads to a rise in the reactive oxygen species (ROS), such as alkoxyl (RO^_^), superoxide (O^•−^_2_), peroxyl (RO_2_·), hydroxyl radicals (OH^•^), and hydrogen peroxide (H_2_O_2_), and reactive nitrogen species (RNS), such as nitrous anhydride (N_2_O_3_), peroxynitrite (ONOO^−^), and nitrogen dioxide (•NO_2_) [41–43]. RNS modifies proteins, leading to protein nitrosylation and the formation of *S*-glutathiols and nitrosothiols. Nitric oxide participates in vascular damage (e.g., BBB and ischemia injury) and inflammatory response by the activation of macrophages, astrocytes, matrix metalloproteinases (MMPs), and adhesion molecules [44, 45]. Peroxynitrites cause the nitration of guanine nucleotides, resulting in DNA single-strand rupture, trigger the PARP pathway, and also prohibit DNA repair enzymes [46]. Nitric oxide inhibits cytochrome *c* oxidase and thereby suppresses mitochondrial ATP production [39]. Hence, excess nitrites accumulation can cause an energy-deficient state in the brain that further ensures the dysfunction of ATP-dependent ion pumps, accumulation of sodium ions (causing cell swelling), increased calcium influx, and hyperexcitability [47]. The augmentation of cytoplasmic calcium levels is the primary mechanism of ROS and RNS output and the activation of calcium-dependent cell demise pathways through the activation of proteases and calpains [48]. In neurodegenerative diseases, free radicals, calcium, lipid peroxidation, and DNA mutilation are at the core of the pathogenic progression of diseases. In previous studies, the analysis of the biomarkers of oxidative stress revealed the amplification of MDA, 4-HNE, and 8-hydroxy-2' -deoxyguanosine (8-OHdG) in the cerebrospinal fluid, brain, and blood samples [49, 50]. In the current experiments, DSM (50 and 100 mg/kg) significantly abrogated QA-ICV-triggered intensification in lipid peroxidation and total nitrites in the mitochondrial portion of the brain. Earlier reports also substantiate the free radical attenuating and anti-inflammatory deeds of DSM in the entire brain of experimental animals [16–20]. The standard drug, DNP, also attenuated the lipid peroxidation (TBARS) and total nitrites in the brain of rats exposed to QA-ICV.

The mitochondria, peroxisomes, endoplasmic reticulum, and plasma membranes are the chief locations of ROS and RNS biosynthesis [51]. Cellular respiration in the mitochondria is the chief generator of ROS, such as superoxide and hydroxyl radicals. Peroxisomes are the central hub for hydrogen peroxide generation. Superoxide anion acquires an electron from molecular oxygen and dismutases to hydrogen peroxide via the Fenton reaction. Subsequently, this hydrogen peroxide is metabolized by catalase to water and oxygen, or it may generate hydroxyl radical. H_2_O_2_ can also generate toxic hydroxyl radicals through the Haber-Weiss reaction. Superoxide anion, by reacting with nitric oxide, can form peroxynitrites [52, 53]. These free radicals trigger mitochondrial permeability transition pore (mPTP), leading to the leakage of cytochrome *c*, mitochondrial degeneration, and cell demise. Mitochondrial complex I is the gateway for electrons' entry from NADH into the respiratory chain. Complex I and II can generate superoxide anions in surplus in response to a higher NADH/NAD^+^ ratio, leading to an abridged FMN (flavin mononucleotide) site on complex I, and electron contribution to the succinate dehydrogenase (SDH)-reduced coenzyme *Q* is associated with a high proton motive force, leading to reverse electron transport [54]. QA is a well-recognized inhibitor of mitochondrial functions [5]. In this study, QA-ICV caused the inhibition of the brain mitofragment complex I and II rates in rats. The QA-induced aberrations in the mitochondrial electron transport chain functions might be the primary cause of oxidative mutilation in the brain of rats. However, DSM (50 and 100 mg/kg) resurrected the brain complex I and II activities in QA-ICV-challenged rats. DNP treatment also showed significant improvement in complex I and II functions in the brain mitochondria against QA-ICV mitotoxicity. The analysis of antioxidant levels revealed that DSM treatment for 21 consecutive days attenuated QA-ICV-induced decline in endogenous antioxidants, such as GSH, GPx, SOD, and catalase. SOD, catalase, and thiol-dependent antioxidants, such as GSH and GPx, are the 1^st^ line of defense against oxidative mutilation. SOD and catalase detoxify superoxide anions and H_2_O_2,_ respectively, and GPx and GSH are involved in the removal of H_2_O_2_ and breakdown of lipid peroxides (MDA, 4-HNE, etc.) to their respective alcohols, particularly in the mitochondria and cytoplasm [55]. In the current study, DSM (50 and 100 mg/kg) or DNP regimens boosted the antioxidant actions in the brain of rats that were rendered toward QA-ICV neurotoxicity.

Neuroprotection by DSM and DNP was evident in the H&E staining analysis of hippocampus and cortical regions of rat brains. QA-ICV treatment caused marked changes in the cellular architecture highlighted by pyknosis, cell swelling, and blebbing of the plasma membrane. These pathogenic changes were attenuated by DSM and DNP treatments in separate groups of rats. In the current protocol, DSM (100 mg/kg) showed significant improvement in biochemical parameters against QA-ICV and also attenuated pathological cell mutilation evident in histological analysis in comparison to DSM (50 mg/kg). Hence, these findings depicted the dose-dependent effects of DSM in the QA-ICV rat model of neurodegeneration.

In the weekly analysis, a significant decline in mean body mass (g), feed, and water ingestion was pragmatic in QA-ICV treated rats. Motor coordination (rotarod test) and gait (footprint analysis) were also adversely affected in rats treated with QA-ICV alone. Locomotor activity was not affected by diverse drug treatments in the current set of experiments. However, DSM or DNP treated rats showed significant improvement in motor coordination and gait against QA-ICV toxicity. Body bulk (g), diet, and water consumption were also enhanced by DSM or DNP in separate groups of QA-ICV treated rats. Memory parameters were assessed using NORT (day 16) and MWM (days 17–21) paradigms. A decline in discrimination ability of QA-ICV treated rats in NORT supported loss of working memory. In MWM trials, QA-ICV caused an increase in ELT during four days training trials and a decrease in TSTQ in retrieval trials on the 5^th^ day. These findings showed the depreciation of long-term spatial memory of rats by QA-ICV treatment. DSM or DNP treatments attenuated the decline of discrimination index, TSTQ, and increase in ELT in rats that were challenged with QA-ICV on day 1. The contemporary findings are in harmony with the former reports substantiating the memory improvement activity of DSM in experimental animals [16–20]. DSM (100 mg/kg) showed dose-dependent improvement in working memory and long-term memory in comparison to DSM (50 mg/kg) against QA-ICV. The findings showed that DSM could ameliorate brain functions such as memory, motor coordination, and gait in rats in the QA-ICV model. Diosmin is a flavonoid glycoside possessing a sugar moiety (rutinoside disaccharide) and aglycone group diosmetin. In several preclinical studies, diosmin is administered through the parenteral route (*i.p.*) [17, 56], and there is an impending urgency to find a suitable formulation to better translate the pre-clinical findings in clinical settings. Particle size reduction and increasing the surface area can be utilized to enhance its transport across the biological barriers [57]. In this context, a micronized diosmin formulation [58] was attempted that showed greater bioavailability and pharmacokinetic characteristics, however, the targeted brain-specific delivery of diosmin is still a huge challenge.

## 5. Conclusions

In the current therapeutic scenario where no such drug is available that can revive the pathogenic evolution of neurodegenerative conditions, such as AD and HD, a shift toward natural remedies is pragmatic. Current therapeutic strategies focus on symptomatic improvement only, and none is able to reverse the sequence of the disease development. We observed that diosmin resurrected cognitive functions (working and long-term spatial memory) in the QA-ICV rat model of neurodegeneration. Diosmin improved the sensorimotor performance and the gait of rats against QA-ICV. The observed improvement of behavioral functions in QA-ICV rats treated with diosmin is primarily because of the attenuation of mitochondrial dysfunctions and oxidative mutilation of the brain. Hence, diosmin might be used as an alternative therapeutic agent against mitochondrial dysfunction origin neurodegenerative disorders. However, additional investigations are mandatory to envisage its neuroprotective mechanism and applications in clinical settings.

## Data Availability

The data of this study are accessible upon an appropriate demand from the corresponding author.
