# Correction to “Mechanistic Insight into Diosmin‐Induced Neuroprotection and Memory Improvement in Intracerebroventricular‐Quinolinic Acid Rat Model: Resurrection of Mitochondrial Functions and Antioxidants”

**DOI:** 10.1155/ecam/9876429

**Published:** 2026-01-29

M. Huang, N. Singh, R. Kainth, M. Khalid, A. S. Kushwah, and M. Kumar, “Mechanistic Insight into Diosmin‐Induced Neuroprotection and Memory Improvement in Intracerebroventricular‐Quinolinic Acid Rat Model: Resurrection of Mitochondrial Functions and Antioxidants,” *Evidence-Based Complementary and Alternative Medicine*, 2022, 8584558, https://doi.org/10.1155/2022/8584558.

In the article, there are errors in Figure 7. Specifically, there are repeated elements between the following panels:

• Sham and QA
• QA + DSM50 and QA + DSM100

The authors explained that this occurred due to the incorrect selection of images during the preparation of the manuscript. Following an assessment of the authors response and the revised figure, the correct Figure 7 is shown below:

**Figure 7:**
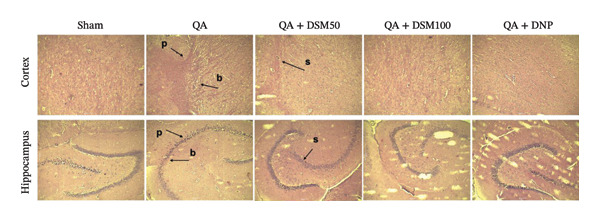
Effect of diosmin (DSM) treatment (doses 50 and 100 mg/kg) on QA‐ICV prompted neurodegenerative deviations in the cortical and hippocampus sections (*n* = 5) (H&E stain, × 40, scale 10 µm). Pyknosis (p), bulging of the plasma membrane (b), and swelling (s) were observed.

We apologize for these errors.

